# Patient’s adherence on pharmacological therapy for benign prostatic hyperplasia (BPH)-associated lower urinary tract symptoms (LUTS) is different: is combination therapy better than monotherapy?

**DOI:** 10.1186/s12894-015-0090-x

**Published:** 2015-09-21

**Authors:** Luca Cindolo, Luisella Pirozzi, Petros Sountoulides, Caterina Fanizza, Marilena Romero, Pietro Castellan, Alessandro Antonelli, Claudio Simeone, Andrea Tubaro, Cosimo de Nunzio, Luigi Schips

**Affiliations:** Department of Urology, “S.Pio da Pietrelcina” Hospital, via San Camillo de Lellis, 1-66054 Vasto, Italy; Department of Clinical Pharmacology and Epidemiology, Fondazione “Mario Negri Sud”, Santa Maria Imbaro, Italy; Department of Urology, General Hospital of Veria, Veria, Greece; Department of Urology, “SS. Annunziata” Hospital, Chieti, Italy; Department of Urology, “Spedali Civili” Hospital, Brescia, Italy; Department of Urology, “Sant’Andrea” Hospital, University “La Sapienza”, Rome, Italy

**Keywords:** Patient adherence, Drug therapy, Benign prostatic hyperplasia, Lower urinary tract symptoms, Alpha blockers, 5alfa reductase inhibitors, Administrative databases, Dutasteride, Finasteride, record-linkage analysis

## Abstract

**Background:**

*Recent studies showed that the non-adherence to the pharmacological therapy of patients affected by BPH-associated LUTS increased the risk of clinical progression of BPH. We* examined the patients adherence to pharmacological therapy and its clinical consequences in men with BPH-associated LUTS looking at the differences between drug classes comparing mono vs combination therapy.

**Methods:**

A retrospective, population-based cohort study, using prescription administrative database and hospital discharge codes from a total of 1.5 million Italian men. Patients ≥40 years, administered alpha-blockers (AB) and 5alpha-reductase inhibitors (5ARIs), alone or in combination (CT), for BPH-associated LUTS were analyzed. The 1–year and long term adherence together with the analyses of hospitalization rates for BPH and BPH-related surgery were examined using multivariable Cox proportional hazards regression model and Pearson chi square test.

**Results:**

Patients exposed to at least 6 months of therapy had a 1-year overall adherence of 29 % (monotherapy AB 35 %, monotherapy 5ARI 18 %, CT 9 %). Patient adherence progressively declined to 15 %, 8 % and 3 % for AB, 5ARI, and CT, respectively at the fifth year of follow up. Patients on CT had a higher discontinuation rate along all the follow-up compared to those under monotherapy with ABs or 5ARIs (all *p* < 0.0001). Moreover, CT was associated with a reduced risk of hospitalization for BPH-related surgery (HR 0.94; *p *< 0.0001) compared to AB monotherapy.

**Conclusions:**

Adherence to pharmacological therapy of BPH-associated LUTS is low and varies depending on drugs class. Patients under CT have a higher likelihood of discontinuing treatment for a number of reasons that should be better investigated. Our study suggests that new strategies aiming to increase patient’s adherence to the prescribed treatment are necessary in order to prevent BPH progression.

## Background

Benign prostatic enlargement (BPE) is caused by a very common histopathological condition in aging men; benign prostatic hyperplasia (BPH). Clinical manifestations of BPH include lower urinary tract symptoms (LUTS), signs and sequelae of bladder outlet obstruction caused by BPE [1]. The prevalence of moderate-to-severe LUTS is high, increasing from 22 % among 50–59 year-old men to 45 % among those in the seventh decade of life. However only 19 % of men suffering from BPH-associated LUTS seek medical treatment and only 10.2 % receive pharmacological treatment [2–4].

Pharmacological therapy for BPH-associated LUTS aims at improving the patient’s quality of life by relieving urinary symptoms and to a certain extent, by preventing the development of BPH-related complications. International guidelines agree that patients with moderate-to-severe LUTS are initially best managed with pharmacological therapy [5, 6].

Five classes of drugs are usually prescribed for the treatment of BPH-associated LUTS: alpha blockers (AB), 5-alpha reductase inhibitors (5ARI), phosphodiesterase-5 (PDE-5) inhibitors, antimuscarinics/beta3 agonists, and phytotherapeutics. Combination therapy (CT) with ABs and 5ARIs has been shown to be beneficial in terms of symptom control and disease progression [7–9].

Although pharmacological treatment of BPH is considered a success story among urologists, daily practice suggests that several patient’s needs remain unmet. Whether or not this is due to drug’s limitations, inappropriate patient management or low patient adherence to drug therapy remains unclear[4,10, 11]. Drug prescription trends and patterns for BPH-associated LUTS show a wide variation amongst countries, this variation is attributed to geographical, societal and cultural differences, cost of medications and different health policies.

A recent paper from our group showed that in “real life” practice the adherence to long-term treatment for BPH-associated LUTS significantly impacts BPH progression [12]. Data on adherence are of importance in order to understand possible unmet needs, explore patient preferences and identify areas for intervention for the health care systems [13]. In this specific issue, data from other areas of medicine confirmed that multiple medications (pills/day) could impact on patient adherence, suggesting that a fixed dose combination (more than a 2-pill therapy) can yield important improvements in patient drug adherence [14].

The aim of this study is to evaluate the patient adherence to pharmacological therapy for BPH-associated LUTS, and to analyze the adherence among drug regimens (CT vs monotherapy) together with long-term effects of drugs discontinuation.

## Methods

A population-based cohort study was conducted using record-linkage analysis of three databases: drug prescriptions database, civil registry and hospital discharge records (HDR) (data on 6.5 million subjects across 22 local Italian Health Authorities).

All Italian citizens have access to health care services; medical and pharmaceutical services are provided for free or at a minimum charge as part of the National Health Service (NHS).

The Italian national drug database includes the prescriptions reimbursed by the Italian NHS; drugs are coded according to the Anatomical Therapeutic Chemical (ATC) Classification [15] and qualified with respect to dosage, and the date of the first and subsequent prescriptions from which data on adherence can be derived.

This cohort was linked with HDR which includes information on primary diagnoses and up to 5 coexisting conditions, performed procedures, dates of hospital admission and discharge. Diagnoses are classified according to the International Classification of Diseases-Ninth Revision, Clinical Modification (ICD9-CM) [16].

The Italian civil registry provides demographic information.

This study’s methodology has been widely used to produce reliable epidemiological surveys [12,17, 18]. The analysis was carried out in strict compliance with the national Italian regulations for the full protection of the privacy rights of the subjects included in the databases. According to the Italian law, no ethical approval is required to perform this type of analysis and no informed consent from patients is needed. LP, CF and MR had the full access to the prescription database.

As reported in the previous paper [12] the sample population consisted of men ≥40 years who had been prescribed medications for BPH-associated LUTS during the index period (January^1st^ 2004-December^31st^ 2006).

Only ABs and 5ARIs were considered in the analysis (ATC codes: G04CA and G04CB, respectively). During the study period, the first prescription of a drug was considered as “index date” for including a patient. Patient adherence to therapy was estimated only for patients receiving treatment for a minimum of 6 months during the index period. Two different levels of exposure to drugs were set: at ≥ 6 months and at ≥12 months. Patients on treatment for more than 12 months during the index period were followed-up for 4 years (median time). Patients who: a) stopped one of the three regimens (AB monotherapy, 5ARI monotherapy or CT) for at least 2 consecutive months during the first year of treatment and at least 4 months/yearly during the follow-up period, or b) switched regimen were considered as “treatment discontinuation”.

Patients were followed until hospitalization or surgery for BPH occurred or until their last follow-up. Patients were excluded when they were diagnosed with urethral stricture, prostate cancer in the 12 months preceding the index day.

Hospital admissions were recorded for patients receiving ≥1 year of pharmacological therapy and they were considered “BPH-related” when hospital records included a primary diagnosis and/or a surgical procedure related to BPH.

The presence of the ICD9-CM 600.xx code as primary diagnosis without surgical procedures was considered as a “BPH-related hospitalization”. In the absence of clear and universally agreed upon indications for BPH-related hospitalization we included in the analyses all the hospitalizations for haematuria, urinary tract infection, urinary retention, bladder stones, and renal failure due to urinary tract obstruction caused by BPH.

The presence of ICD9-CM codes 57.0,57.91,57.92,60.21, and 60.29,60.3,60.4 as primary or secondary surgical procedures with any primary diagnoses was considered hospitalization for “BPH-related surgery”.

### Statistical analysis

For patients with at least 12 months of treatment, the characteristics were reported using descriptive statistics. Differences between patient treatment subgroups were assessed using a standardized difference (SD). Crude incidence rates (IRs) per 1000 men/year and incidence rate ratios (IRRs) with 95 % confidence intervals (CIs) were calculated with the Poisson regression model.

A multivariable Cox proportional hazards regression model was used to account for differences in follow-up and in baseline characteristics among groups. In all Cox models, the associations between groups and all outcomes were adjusted for co-variates known to be of prognostic importance to the outcomes: age and previous hospitalization for BPH, history of BPH-related surgery, and previous pharmacological treatment. Results were expressed as hazard ratios (HRs) and 95 % CIs. Adjusted event-free survival curves were calculated using the corrected group prognosis method. Discontinuation rate according to treatment group was compare using Pearson chi square test.

All reported that *p*-values are two tailed, and a *p*-value less than 0.05 was considered statistically different.

Analyses were conducted using SAS Statistical Package Release9.3 (SAS Institute, Cary, NC, USA).

## Results

From the initial cohort of about 6.5 million individuals, men ≥40 years old were 1,447,074. Among these, only 28,273 received prescriptions for 12 months for BPH-associated LUTS, and this group was followed (median follow up 4 years, IQR 2–5.3) and represented our study cohort.

General characteristics of the group are summarized in Table 1. ABs was the most frequently prescribed drug class (87.1 %), followed by 5ARIs (8.1 %) and CT (4.7 %) that was prescribed in older patients.

**Table 1:** Patients’ characteristics in relation to BPH treatment.

Variable	Baseline					
	Overall	AB	5ARI	CT	% Standardized difference *	
	28273	24626	2309	1338	5ARI vs AB	CT vs AB
	No. (%)	No. (%)	No. (%)	No. (%)		
*Mean age (± SD)*	70.28 (9.46)	69.55 (9.36)	75.61 (8.72)	74.49 (8.25)	66.99	58.3
*Age*						
40–55	1699 (6.01)	1641 (6.66)	42 (1.82)	16 (1.20)	−24.22	−28.42
56–65	7001 (24.76)	6579 (26.72)	248 (10.74)	174 (13.00)	−41.83	−34.89
66–75	11,120 (39.33)	9819 (39.87)	791 (34.26)	510 (38.12)	−11.65	−3.6
76–85	7054 (24.95)	5555 (22.56)	961 (41.62)	538 (40.21)	41.71	38.75
>85	1399 (4.95)	1032 (4.19)	267 (11.56)	100 (7.47)	27.63	14.04
* Previous hospitalization for BPH*	1312 (4.64)	1048 (4.26)	167 (7.23)	97 (7.25)	12.82	12.88
* Previous BPH surgery*	98 (0.35)	88 (0.36)	7 (0.30)	3 (0.22)	−0.94	−2.47
* Previous BPH severity factors*	854 (3.02)	715 (2.90)	95 (4.11)	44 (3.29)	6.58	2.22
* Previous BPH related therapy*	16,491 (58.33)	14,220 (57.74)	1377 (59.64)	894 (66.82)	3.84	18.8

Legend: AB: Alpha-blocker monotherapy; CT: Combination Therapy 5ARI; 5-alpha reductase inhibitors monotherapy; * Standardized difference greater than 10 % represents meaningful imbalance in explored variable between treatment groups

### Drug adherence

**Fig. 1:**
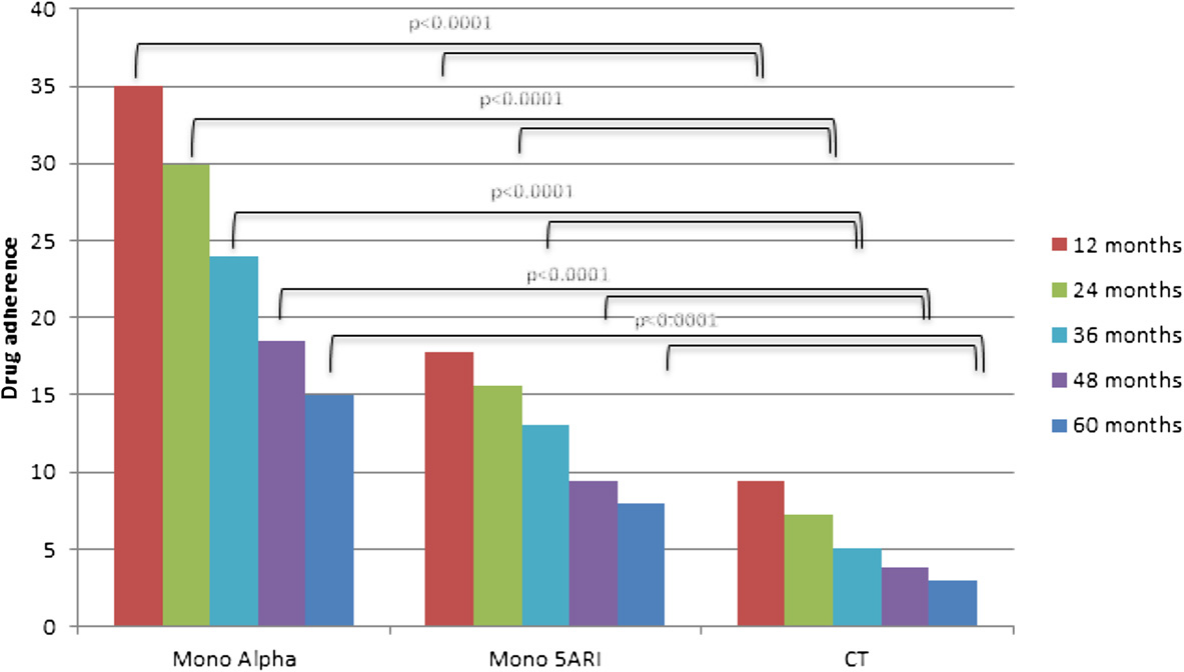
Differences in adherence between different pharmacological regimens at 1, 2, 3, 4 and 5 years of follow-up

Patients who received prescriptions for ≥6 months were 97,407, and decreased to 61298 (63 %) at 10 months and to 28,273 (29 %) at 12 months. Patients who continued taking their drugs for up to 12 months were 35 %, 18 % and 9 % for ABs, 5ARIs and CT, respectively. These rates decreased to 15 %, 8 % and 3 % at 60 months (Fig. 1).

In men who received drug prescription for at least 12 months, the 5-year adherence was 42 % (Fig. 1). Patients who remained under pharmacological therapy for the entire follow-up period (median 4 years) represented 13 % of those identified in the index period.

Patients under CT showed a higher discontinuation rate all along the follow-up (*p *< 0.0001) compared to either monotherapy (Fig. 1).

During the follow-up period, only 12270 patients continued their prescribed pharmacological therapy. The discontinuation rate was statistically significant higher in patients under CT (discontinued *vs* adherent patients: SD % 19.87) (Table 2).

**Table 2:** Patients’ characteristics according to drug adherence

VARIABLE	Discontinuated patients	Adherent patients	Standardized difference (%) *
*Mean age (± SD)*	70.15 (9.6)	70.37 (9.34)	-
Age class			
40–55	762 (6.21)	937 (5.86)	−1.4916
56–65	3131 (25.52)	3870 (24.18)	−3.0886
66–75	4784 (38.99)	6336 (39.59)	1.2350
76–85	2936 (23.93)	4118 (25.73)	4.1775
>85	657 (5.35)	742 (4.64)	−3.2957
* Previous hospitalization for BPH*	560 (4.56)	752 (4.70)	0.6430
* Previous BPH surgery*	45 (0.37)	53 (0.33)	−0.6030
* Previous BPH severity factors*	392 (3.19)	462 (2.89)	−1.7928
* Previous BPH related therapy*	7155 (58.31)	9336 (58.34)	0.0529
*Therapeutic regimen*			
* AB*	10923 (89.02)	13703 (85.63)	−10.2158
* 5ARI*	1050 (8.56)	1259 (7.87)	*−2.5140*
* CT*	297 (2.42)	1041 (6.51)	*19.8785*

Legend: AB: Alpha-blocker monotherapy; 5ARI; 5-alpha reductase inhibitors monotherapy; CT: Combination Therapy; * Standardized difference greater than 10 % represents meaningful imbalance in explored variable between treatment groups

### Hospitalization rates

During the follow-up period, the hospitalization rates for BPH and BPH-related surgery were 9.04 (95 % CI 8.49–9.62) per 1000 patient/year and 12.6 (95 % CI 11.96–13.28) per 1000 patient/year, respectively (Table 3).

**Table 3:** Hospitalization rates for BPH and BPH-related surgery

Outcomes	Overall		Mono alpha		Mono 5ARI		CT	
	Events	IR (95 % CI)	Events	IR (95 % CI)	Events	IR (95 % CI)	Events	IR (95 % CI)
Hospitalization for BPH (non surgical reasons)	989	9.04 (8.49;9.62)	918	9.58 (8.98;10.22)	34	3.77 (2.69;5.27)	37	8.10 (5.87;11.18)
BPH - related surgery	1393	12.60 (11.96;13.28)	1351	13.96 (13.23;14.72)	23	2.54 (1.69;3.82)	19	4.08 (2.60;6.40)

Legend: AB: Alpha-blocker monotherapy; 5ARI; 5-alpha reductase inhibitors monotherapy; CT: Combination Therapy; IR: incidence rate for 1000 person-years

As previously shown [12], the multivariate analysis confirmed that the use of 5ARIs was associated with a reduced risk of hospitalization due to BPH and BPH-related surgery (HR 0.46, 95 % CI 0.33–0.65 and HR 0.23, 95 % CI 0.15–0.35; *p* < 0.0001).Drug discontinuation on multivariate analysis was an independent risk factor for either BPH-related hospitalization or BPH surgery regardless of the therapeutic group (HR 1.65, 95 % CI 1.43–1.89 and HR 2.80, 95 % CI 2.59–3.03; *p* < 0.0001), as already reported [12].

## Discussion

BPH represents a major public health issue because of its increasing prevalence, progressive nature and treatment costs [19–21]. Current guidelines recommend the use of ABs and 5ARIs as monotherapy or in combination for the treatment of BPH-associated LUTS [5,6]. However, a gap exists between guidelines and actual clinical practice [10,12, 21]. In “real life” the low adherence to prescribed medications is a recognized problem for chronic diseases [13]. Some studies deeply evaluated the problems of drug prescription and adherence for BPH as well as its impact on the clinical outcomes [12, 19, 21–23]. All showed concordant results: 1) the reported adherence in clinical trials is higher than that observed in real life; 2) the duration of treatment for BPH-associated LUTS is extremely short; 3) the adherence to treatment is generally low and 4) this might negatively influence BPH-related hospitalization rates.

By and large, patient adherence, or compliance, to a prescribed drug treatment is defined as the extent to which a person's attitude in terms of taking medication coincides with the medical or health advice he receives.

Adherence or compliance to a drug regimen is divided to primary non-compliance, for example when one receives a prescription, but does not have it made up at a pharmacy. Forms of secondary non-compliance include taking incorrect doses of the prescribed medication, taking the medication at wrong times, forgetting one or more doses of the medication, or altogether stopping the medication, either by ceasing to take the medication sooner than the doctor recommended or failing to obtain a repeat prescription [24]. Poor adherence to a therapeutic regimen has been identified as a major public health problem that may have a major impact on clinical outcomes [25].

The lack of a valid method for measuring compliance is by itself a major barrier to compliance research. Both direct and indirect measures have been sought in order to quantify compliance, and although direct measures are considered to be the most accurate, their invasive nature makes them unacceptable and inappropriate to use. Indirect measurements are therefore more frequently reported in the literature and include measures such as interviews, diaries, tablet counts, and prescription refill dates. Interviews and all self-report methods are vulnerable to overestimates of compliance and underestimates of non-compliance [26]. There are inherent limitations with these methods for generating valid and reliable data to give an accurate estimate of extent of patient adherence. Data from administrative databases, used in our and in other studies, are another indirect but reliable method of estimating drug consumption and patient adherence to a certain regimen [12, 21, 22]. Patient adherence to medical treatment is generally suboptimal irrespective of the drug or the treated condition. Studies on patient adherence on pharmacological therapy for the treatment of hypertension have shown that 50 % of patients discontinue their medication while two-thirds of patients that stay on treatment seem to lower their drug doses [26].

Although data regarding pharmacotherapy for BPH-associated LUTS are limited, a recent study from France confirmed that the 1-year adherence ranges between 21 % to 26 [21]. Madershbacher in 2007, looking at the results from clinical trials, depicted a clinical scenario describing the adherence rates of different therapeutic regimens. They found that the discontinuation rates in the 5-ARIs trials were lower than in the AB trials; and confirmed a 2-year discontinuation rate of 10–20 % of patients under 5ARIs. On the other hand, discontinuation rates were lower for combination therapy (18 %) compared to finasteride (24 %) and doxazosin (27 %) monotherapy [19]. Even more interesting, these data are in contradiction with our results: a statistically significant lower adherence for CT compared to either AB or 5ARI monotherapies. These discrepancies are probably due to different study design. Our “real life” approach shows that patients abandon CT for several reasons that should be better investigated. Moreover, we found that the prolonged use of 5ARIs and adherence to the prescribed regimen were significantly associated with a lower risk of BPH-related hospitalization and surgery.

In the BPH population the decision to adhere to pharmacological treatment is primarily based on the patient’s perception of bother due to LUTS and its impact on quality of life, and definitely depends on patient expectations and beliefs. The patient’s perspective towards BPH and its management play a major role in the decision to initiate, continue or abandon treatment [27]. Even if we recognize that the reasons for the lack of drug adherence are multiple and difficult to analyze (especially using this methodological approach), it is important to remember that the strategy “enhance compliance by decreasing the number of pills” has been widely demonstrated in other fields of medicine [14]. There is convincing evidence from the literature suggesting that adherence is inversely associated with the complexity of the drug regimen. In this concept, the so-called fixed-dose combination (FDC) drugs (2 or more drugs produced in a single pill/tablet) have been developed in order to treat one disease with complementary actions (e.g., diabetes mellitus, asthma) or treat multiple clinical conditions (e.g., hypertension and hyperlipidemia) [14]. A FDC regime containing 0.5 mg of dutasteride and 0.4 mg of tamsulosin in the same pill is available for the treatment of BPH-associated LUTS.

Recent reports show that the use of FDC is associated with lifestyle advice resulted in rapid and sustained improvements in men with moderate BPH-associated LUTS [28] and that FDC is a cost-effective option in a estimated lifetime budget cost model [29] .

Even though the parameters that would modify patient’s adherence are multiple and complex (spanning from awareness campaigns to better patient counselling) it seems reasonable to support the use of FDC for BPH-associated LUTS in order to decrease patient withdrawal and to increase adherence to the guidelines.

Several limitations of our study should be acknowledged. Studies based on data from administrative databases cannot be considered efficacy studies and do not include clinical variables or patient reported outcomes [30]. Another serious limitation is the imbalance between regimens, but this reflects the prescription attitudes. Moreover the current analysis is specific to the Italian situation and its generalization should be done with caution.

In this study we demonstrated that in “real life” patient adherence to BPH medication is different to that reported in clinical trials and that patients under CT abandon treatment more frequently that patients under monotherapy do.

## Conclusions

Patient adherence to pharmacotherapy for BPH-associated LUTS is low. The need for combining two drugs to treat BPH represents a serious obstacle to better adherence. Persistence on pharmacological treatment is associated with a lower rate of hospitalization for BPH-related reasons. The use of a fixed-dose combination drug could increase adherence to treatment and would likely prevent BPH progression.

## Abbreviations

5ARIs: 5alpha-reductase inhibitors
AB: Alpha-blocker
ATC: Anatomical Therapeutic Chemical
BPE: Benign prostatic enlargement
BPH: Benign prostatic hyperplasia
CIs: Confidence intervals
CT: Combination therapy
FDC: Fixed-dose combination
HDR: Hospital discharge records
HRs: Hazard ratios
ICD9-CM: International Classification of Diseases-Ninth Revision, Clinical Modification
IRRs: Incidence rate ratios
IRs: Incidence rates
LUTS: Lower urinary tract symptoms
NHS: National Health Service
PDE-5: Phosphodiesterase-5
SD: Standardized difference
